# Nutritional Characterisation of *Acheta domesticus* at Different Developmental Stages for Application in Animal Feed

**DOI:** 10.1111/jpn.70085

**Published:** 2026-07-16

**Authors:** Mariarosaria Lanzieri, Marcello Scivicco, Nicola Francesco Addeo, Simone Vozzo, Lorella Severino, Rosario Davino, Dolores Ianniciello, Carmen Scieuzo, Rosanna Salvia, Angela De Bonis, Patrizia Falabella, Fulvia Bovera

**Affiliations:** ^1^ Department of Veterinary Medicine and Animal Production University of Naples Federico II Napoli Italy; ^2^ Department of Basic and Applied Sciences University of Basilicata Potenza Italy; ^3^ Spinoff XFlies s.r.l. University of Basilicata Potenza Italy

**Keywords:** *Acheta domesticus*, developmental stage, insect‐based feed, nutritional composition, protein digestibility

## Abstract

The house cricket *Acheta domesticus* has emerged as a promising alternative protein source for animal nutrition; however, limited information is available on how developmental stage influences its nutritional characteristics. This study compared subadults (30‐day‐old) and adults (40‐day‐old) crickets in terms of chemical composition, mineral profile and heavy metal‐content, fatty acid profile, crude protein digestibility, and chitin yield. Adults showed significantly higher dry matter, crude protein, nitrogen‐free extract, and protein digestibility values, whereas subadults exhibited ether extract and ash content, resulting in higher gross energy. Mineral analysis revealed low levels of toxic elements in both groups, with adults displaying higher selenium and phosphorus concentrations. Fatty acid profiling highlighted marked developmental differences: subadults had higher MUFA and linoleic acid, alongside an unbalanced ω6/ω3 ratio, while adults exhibited a more favourable fatty acid distribution, including increased long‐chain ω3 and ω6 derivatives such as arachidonic acid. Chitin extraction revealed no significant differences between groups, although a strong negative correlation was identified between chitin content and crude protein digestibility. Overall, the results indicate that while adults provide superior protein quality and a more balanced lipid profile, subadults offer advantages in terms of energy content and potentially reduced production time. These findings support the strategic integration of *A. domesticus* at different developmental stages into animal feed formulations and underscore the importance of stage‐specific nutritional characterisation for optimising insect‐based feed ingredients.

## 1 Introduction

The growing global demand for sustainable food/feed production has introduced significant challenges for the livestock sector (FAO 2019). Driven by a rapidly increasing world population and the corresponding rise in animal protein consumption, there is an urgent need to reassess conventional protein sources such as soybean meal and fishmeal, which are increasingly constrained by environmental, agronomic, and economic limitations (FAO 2019). Fishmeal, once a staple in animal nutrition due to its high digestibility and balanced amino acid profile, has seen a progressive decline in use primarily attributed to the variability in its nutritional quality, potential risks of pathogen contamination, and mounting environmental concerns (Khan 2018; Gasco et al. 2019; Choidovà and Tùmovà 2020). Soybean meal has emerged as the dominant protein source; however, it presents notable limitations, including a deficiency in methionine, an essential amino acid, and agricultural practices that may negatively impact on the environment (Khan 2018; Gasco et al. 2019). In response to these limitations, edible insects have garnered increasing attention as a viable and sustainable protein alternative. As interest in innovative and sustainable food/feed solutions grows among researchers, industry stakeholders, and consumers alike, insects continue to emerge as promising components of future food and feed systems (Kim et al. 2018). In parallel, the development of reliable species authentication methods has become increasingly important to ensure the traceability and integrity of insect‐derived products, particularly as their role in commercial feed and food applications expands (Fernandez‐Cassi et al. 2019). Their nutritional composition and potential role within more sustainable production systems position them as promising candidates for inclusion in animal feed (Ververis et al. 2022; EFSA et al. 2024). Among these, the house cricket (*Acheta domesticus*, Linnaeus, 1758; Orthoptera: Gryllidae) has been extensively studied due to its high protein content, balanced amino acid profile, and suitability for use in processed forms such as meal or powder for feed applications (Kulma et al. 2019; Spano et al. 2023). The regulatory framework in the European Union has progressively adapted to this innovation. Since 2017, processed animal proteins (PAP) derived from eight insect species, including *A. domesticus*, were authorised for use in aquafeed (Commission Regulation (EU) 2017/893; 2021/1925). This was expanded in 2021 to include their use in pig and poultry diets (Commission Regulation (EU) 2021/1372). The nutritional potential of *A. domesticus* supports its inclusion as a complementary protein source in animal feed, contributing to the diversification of feed resources and helping to reduce reliance on conventional ingredients such as soybean meals, rather than serving as a direct replacement (Cullere et al. 2019). However, their sustainability advantage largely depends on the use of low‐value organic side streams that are not directly suitable for livestock feeding, thereby contributing to a more efficient use of available resources. Its integration into broiler and layer hen diets aligns with broader shifts in poultry production toward slower‐growing breeds and outdoor rearing systems, which are increasingly favoured by consumers for perceived improvements in animal welfare and product quality (Nieto et al. 2024). In such systems, the demand for digestible and high‐quality protein sources is particularly pronounced (Gasco et al. 2019). However, the nutritional efficacy of *A. domesticus* proteins can vary significantly depending on several biological and management factors including age. In *A. domesticus*, the transition from subadults to adults is closely linked to age and developmental stage. Subadult crickets are typically individuals of about 30 day‐old while adulthood is generally reached at about 40 days, marked by sexual maturity and completion of morphological development (Liao et al. 2025). Overall, both adults and subadults are rich in protein and other nutrients, but their nutritional profiles differ, making developmental stage an important factor in cricket food production and ingredient selection. Therefore, targeted evaluations are essential to guide the controlled and rational incorporation of *A. domesticus* into feed formulations (Kulma et al. 2019; Ververis et al. 2022; Pedrazzani et al. 2024). House crickets are usually harvested during the last instar, right before or right after ecdysis into adults, because they gain approximately half of their total weight during the last instar (Veldkamp et al. 2021; Kyllonen and Manzanares 2022). However, for sustainable use in animal feed, it could be interesting to evaluate if developmental stages preceding the last moult may present overlapping chemical‐nutritional characteristics, with the advantage of reducing production times and costs.

From an animal nutrition perspective, understanding how developmental stage affects protein quality, lipid composition and mineral profile is essential for the rational use of *A. domesticus* meals in feed formulation and ingredient selection. Beyond their nutritional value, monitoring the content of heavy metals is essential to ensure the safe use of insect flour. Cadmium (Cd), lead (Pb), mercury (Hg), and arsenic (As) are priority contaminants in feed because they can bioaccumulate and exert toxic effects even at low concentrations (EFSA 2026). Recent studies have shown that species such as *A. domesticus*, *Tenebrio molitor*, and *Locusta migratoria* can contain heavy‐metal residues at levels that in some cases exceed regulatory limits (Ramos et al. 2024; San Onofre et al. 2024). In the European Union, the maximum permissible levels in feed materials are defined by Directive 2002/32/EC and Commission Regulation (EU) 2019/1869, which set maximum residues of 0.5 mg/kg for Cd, 5 mg/kg for Pb, 0.1 mg/kg for Hg, and 2 mg/kg for As. Assessing these elements is therefore essential to ensure that novel protein sources such as *A. domesticus* comply with feed and food safety standards.

Thus, this study aims to analyse the chemical composition, mineral profile, heavy‐metal content, fibre content, fatty acid profile, crude protein content and digestibility of *A. domesticus*, both adults and subadults, with the aim of evaluating its potential as an alternative protein source in animal feed.

## 2 Materials and Methods

### 2.1 Insect Rearing and Processing

Sixteen samples of *A. domesticus*, eight adults (40‐day‐old) and eight subadults (30‐day‐old), obtained from eight different production cycles throughout 2024, were provided by Evolveat, based in Camerata Picena (AN), Italy. Each individual sample represented one biological replicate. For both life stages, the same rearing substrate was used. The chemical characteristics and fatty acid profile are reported in Table 1. The rearing conditions included the use of 60 × 40 × 40 cm plastic tanks, each containing 6000 specimens, in a vertical rearing system. The environmental temperature was maintained at 30.5°C with a relative humidity of 60%. Feed was administered every ten days in quantities of approximately 1 kg per tank; water was supplied via drinkers at the same frequency and in the same amount. Inside the tanks, cardboard structures (egg cartons of 30 × 30 × 5 cm) were used as environmental enrichment. At 30 days (subadults) and 40 days (adults), crickets were separated from the substrate by using specific collectors (commercial egg cartons) exposed to light, to exploit the natural phototaxis of insects. Once collected, the crickets were subjected to a starvation phase, during which residual food and feeders were removed for at least 24 h, in order to empty the digestive tract and then to an automatic sieving to mechanically separate the animals from residual substrate, frass, and other debris. Subsequently, the crickets were subjected to rapid cooling by means of a blast chiller at −22°C for 24 h. After this phase, they were washed twice with drinking water in combination with food‐grade ethanol (C_2_H_5_OH) in a ratio 80/20, in order to remove surface impurities. Excess washing liquid was removed by centrifugation (Eppendorf, Hamburg, Germany), in order to facilitate effective elimination of residual humidity. Subsequently, heat treatment was carried out in an electric oven (Memmert, Germany), bringing the core temperature of the product up to at least 80°C for at least 2 h. This step was applied as a laboratory‐scale treatment to reduce microbial load and obtain a stable product. Finally, the crickets were ground with an electric mill (IKA‐Werke GmbH & Co. KG, Staufen, Germany), obtaining flour. The meal obtained was manually sieved in two phases using 100 and 50 US mesh sieves, corresponding to particle sizes of approximately 150–300 µm. Sieving was performed after complete drying of the samples to reduce fat‐related stickiness and prevent clogging and was carried out at room temperature without additional cooling treatments.

**Table 1:** Chemical characteristics and fatty acid profile of the substrates utilised as feed for *Acheta domesticus* growth.

Chemical‐nutritional characteristics	
Moisture, %	7.99
Crude Ash, % DM	8.01
Crude Lipids, % DM	5.76
Crude Protein, % DM	25.34
Crude Fibre, % DM	7.27
Nitrogen‐free extract, % DM	53.60
Gross Energy (Kcal/kg)	4738
**Fatty acid profile, g FA/100 g total FAME**	
C4:0	0.15
C12:0	0.13
C14:0	0.20
C16:0	10.85
C16:1	0.31
C18:0	2.51
C18:1 trans 9	0.12
C18:1 cis 9	31.40
C18:2 cis ω6	52.90
C20:0	0.44
C18:3 ω3	2.07
C20:2 ω6	0.02
C22:0	0.13
C22:1	0.05
C24:0	0.15
C20:5 ω3	0.02
SFA	14.56
MUFA	31.88
PUFA	55.00
ω6	52.92
ω3	2.09
PUFA/SFA	3.78
ω6/ω3	25.38
LA/ALA	25.58

*Note:* Ingredients: soybean meal, corn, toasted soybeans, wheat middlings, sunflower meal, corn germ meal, wheat semolina, wheat bran, calcium carbonate, cane molasses, monocalcium phosphate, sodium chloride, potassium carbonate, magnesium sulphate monohydrate, algae‐based calcium (Lithothamnium), magnesium sulphate heptahydrate, inactive yeast from Saccharomyces cerevisiae, magnesium carbonate, calcium sulphate, sulphur flowers, and wheat. Nutritional additives: Vitamin A 12.500IU, Vitamin D3 5.000IU, Vitamin E 62.50 mg, Vitamin B1 13.8 mg/kg, Vitamin B2 12.6 mg/kg, Vitamin B6 2.9 mg/kg, Vitamin B12 0.15 mg/kg, Vitamin C 350 mg/kg, Niacianamide 315 mg/kg, Biotin 0.79 mg/kg, Choline chloride 1.054 mg/kg, Folic acid 0.029 mg/kg, Calcium d‐Pantothenate 28.2 mg/kg, Beta‐carotene 5.0 mg/kg. Trace element compounds: Ferrous sulphate monohydrate 120 mg/kg, Potassium iodide 5.6 mg/kg, Manganese oxide 158 mg/kg, Copper sulphate pentahydrate 28.9 mg/kg, Sodium selenite 0.97 mg/kg, Zinc oxide 151 mg/kg, Zinc sulphate monohydrate 140 mg/kg. Antioxidants: Tocopherol extracts from vegetable oils 50 mg/kg. Binders: Sedimentary origin clinoptilolite 78.1 mg/kg.

Abbreviations: AA, Arachidonic acid; ALA, Alpha‐linolenic acid; EPA, Eicosapentaenoic acid; LA, linoleic acid; MUFA, monounsaturated fatty acids; PUFA, polyunsaturated fatty acids; SFA, saturated fatty acids.

### 2.2 Chemical Composition

Chemical composition analyses of rearing substrate, subadults and adult samples were carried out in triplicate, in accordance with the official AOAC methods (AOAC 2004): dry matter (DM, method 943.01), ash (method 924.05), crude protein (CP, method 954.01), ether extract (EE, method 920.39), crude fibre (CF, method 962.09), acid detergent fibre (ADF, method 973.18). CP content was estimated using different nitrogen‐to‐protein conversion factors: 6.25 for diet samples and 5.0 for cricket samples, due to the presence of non‐protein nitrogen associated with chitin (Ritvanen et al. 2020; Mitchaothai et al. 2022). The Nitrogen‐Free Extracts (NFE) were calculated as follows: NFE (% NFE) = 100 – [CP (% DM) + EE (% DM) + CF (% DM) + Ash (% DM)]. Gross energy was estimated from proximate composition using the predictive equation proposed by Song et al. (2025): GE (kcal/kg DM) = 4341 + 11 × CP + 54 × EE −24 × ash.

### 2.3 In Vitro Digestibility of Crude Proteins

The CP digestibility of insect samples was evaluated by an in vitro enzymatic assay based on pepsin digestion, according to Mertz et al. (1984). The method simulates gastric digestive conditions of monogastric animals using pepsin digestion.

All samples were gently ground and sieved through a 1 mm sieve to ensure sample homogeneity and remove any aggregates prior to in vitro digestion testing. They were then weighed (0.2 g ± 0.01 g) into 150 mL glass tubes without prior degreasing. A phosphate buffer solution at pH 2.0 was prepared by dissolving 6.8 g of KH_2_PO_4_ and 7.1 g of Na_2_HPO_4_ in distilled water to a final volume of 1 L. The pH was adjusted with HCl 1M. The buffer solution was used to dissolve pepsin (≥ 2500 units/mg protein (E1%/280)) at a concentration of 10 mg/mL. For each sample, 20 mL of this pepsin solution were added. The enzyme used was of porcine origin and supplied by Sigma‐Aldrich (St. Louis, MO, USA). Samples were incubated at 37°C for 2 h in a heated bath with continuous stirring. At the end of digestion, the test pieces were immersed in cold water (4°C) for 30 min to block enzyme activity. The samples were then centrifuged at 4000 rpm for 15 min. The supernatant was removed; the pellets washed with 10 mL of phosphate buffer pH 7.0 and centrifuged again under the same conditions. The solid residues were filtered on Whatman paper No. 401, dried at 100°C for 15 min and then analysed for CP content according to the Kjeldahl method (AOAC 2004 method number 954.01).

The in vitro digestibility coefficient was obtained by the following formula by Murugu et al. (2021):

$$\mathrm{CPd}=\frac{\left[\mathrm{CPs}-\mathrm{CPr}\right]}{\mathrm{CPs}}\times 100$$

Where:

CPd is the digestibility of the crude protein; CPs is the crude protein content of the samples; CPr is the crude protein content of the residual material after digestion.

### 2.4 Mineral Profile

Two aliquots (A and B) of each insect sample (0.5 g ± 0.02) were placed in Teflon vessels and mixed with 7.0 mL of 65% HNO_3_ and 3.0 mL of 30% H_2_O_2_ (Romil UpA, Romil Ltd., Cambridge, UK). The vessels were placed in a microwave digestion system (Milestone Inc., Controls Drive, Shelton, CT 06484). Wet digestion in the microwave was performed with a digestion programme for 60 min at 190°C. Each vessel was then cooled to 30°C and the digestion mixture was transferred to a 50.0 mL flask. The final volume was obtained by adding Milli‐Q water. The mineralised samples were then filtered using 0.45 µm SY25TF (PTFE) filters. Analysis of total As, Pb, Hg, Cd, Se, Fe, Ca, Mg, K and P levels was performed using an AA‐6300 spectrophotometer (Shimadzu, Columbia, MD, USA) equipped with an ASC‐6100 autosampler (Shimadzu, Columbia, MD, USA) and a GFA‐EX7i graphite furnace atomiser (Shimadzu, Columbia, MD, USA). The Multi‐Element Programme Software 4.3 was used for data processing. Argon was used as both internal and external gas, with a hollow cathode lamp for element‐specific emissions and a deuterium lamp for background correction. Lamp intensity, sample injection volume, and temperature ranges (1600°C–1800°C for atomisation) were varied to optimise the analytical signal. Detection of As, Pb, Hg, Cd, Se, Fe, Ca, Mg, K and P was performed by atomic absorption spectrophotometry according to the method of AOAC International (1995), using the indicated equipment and software as described.

### 2.5 Fatty Acid Profile

The fatty acid profile of rearing substrate and insects was determined after lipid extraction by the method of Folch et al. (1957). The lipid extracts were initially gravimetrically quantified, then saponified and methylated for analysis of fatty acid content by gas chromatography, model TRACE 1310 (Thermo Scientific SpA, Rodano, Milan, Italy) equipped with an AI 1310 Autoinjector/AS 1310 Autosampler. The gas chromatograph was equipped with a flame ionisation detector (FID). To quantify fatty acid methyl esters (FAMEs), we used an internal standard (C23:0; Supelco, Bellefonte, PA, USA) and an external standard (37‐component FAME mixture Supelco; Supelco, Bellefonte, PA, USA) necessary to compare FAME retention times and obtain calibration curves.

### 2.6 Chitin Extraction

Chitin was extracted from subadults and adult cricket flour, following the procedure described by Triunfo et al. (2022), with minor modifications. For each sample, 10 g of flour was used after sieving to remove the larger particles. The samples were first defatted using petroleum ether in a 1:10 (w/v) ratio by stirring at room temperature (RT) for 50 min and then heating to 80°C for 10 min. The defatted powder was filtered, left under a fume hood for 24 h to allow the solvent residues to evaporate, and then demineralised with 0.5 M formic acid (1:10 w/v) for 1 h at RT. The resulting samples were then subjected to deproteinization reaction with 2 M NaOH (1:10 w/v) for 2 h at 80°C (Triunfo et al. 2022; Franco et al. 2021; Tzompa‐Sosa et al. 2014). After each treatment, the material was filtered, thoroughly washed with distilled water until neutrality, and dried in an oven at 60°C until a constant weight was reached. The percentage by weight of lipids and proteins removed during each step was calculated on samples dry weight according to Equations (1) and (2). The final chitin content was determined using Equation (3).

(1) $$\%\;\mathrm{lipids}\;\mathrm{removed}=\frac{W_{\mathrm{raw}}-W_{\mathrm{defat}}\;}{W_{\mathrm{raw}}}\;*\;100$$

(2) $$\%\;\mathrm{proteins}\;\mathrm{removed}=\frac{W_{\mathrm{demin}}-W_{\mathrm{deprot}}\;}{W_{\mathrm{raw}}}\;*\;100$$

(3) $$\%\;\mathrm{extracted}\;\mathrm{chitin}\;(\mathrm{yield})=\frac{W_{\mathrm{deprot}}\;}{W_{\mathrm{raw}}}\;*\;100$$

Where *W*_*raw*_ is the weight of the starting sample; *W*_*defat*_ is the weight after defatting process; *W*_*demin*_ is the weight after demineralisation; and *W*_*deprot*_ is the weight after deproteinization (the final residue, i.e., chitin).

### 2.7 ATR‐FTIR Analysis

The infrared spectra were acquired in the 400–4000 cm^−1^ range by utilising a Bruker Alpha II spectrophotometer in the ATR configuration. To achieve 2 cm^−1^ of spectral resolution 24 scans were conducted.

### 2.8 Statistical Analysis

Data on chemical composition, CP digestibility, mineral profile and fatty acid profile were analysed by one‐way ANOVA using the PROC GLM procedure of SAS (1974) (SAS Institute Inc., Cary, NC, USA), considering developmental stage (subadults vs. adults) as the fixed effect, according to the following model:

Yij=μ+Gi+εij

where Yij is the observed value, μ is the overall mean, Gi is the fixed effect of developmental stage, and εij is the residual error.

Data from chitin extraction were expressed as mean ± standard deviation (SD). Their distribution was assessed by the Shapiro–Wilk test using GraphPad Prism version 8.4.2 for Windows (GraphPad Software, San Diego, CA, USA). When data were normally distributed (*p* > 0.05), comparisons between subadults and adults were performed using an unpaired Student's *t*‐test; otherwise, the Mann–Whitney U test was applied. The relationship between chitin content and CP digestibility was evaluated by Pearson's correlation and linear regression analysis. Differences were considered statistically significant at *p* < 0.05.

## 3 Results

Table 2 shows the results of chemical analysis and CPd of *A. domesticus*, comparing adults and subadults. Only ash and ether extract percentages (*p* < 0.01) and calculated gross energy content (*p* < 0.05) were higher in the subadults, while all the other parameters reported in the table showed significantly higher values in adults. The content of toxic elements in subadults (SA) and adult (AD) samples of *A. domesticus* was presented in Table 3. Arsenic data were not shown in the table because its levels were below the limit measurable by the instrument (< 0.001 mg/kg). The SA samples showed higher values of Cd (*p* < 0.05) and Hg (*p* < 0.01) than the adults, while no differences were found for the levels of lead. Table 4 shows the concentration of macro and micro elements in *A. domesticus* subadults and adults. The adult stage showed the highest levels of Se and P (*p* < 0.01). Table 5 shows the analysis of the fatty acid profile of *A. domesticus*, revealing several significant differences between adults and subadult individuals. In adults, a significantly higher content of short and medium chain acids such as C4:0 and C10:0 was observed (*p* < 0.05), but also of long chain acids such as C22:0 and C24:0 (*p* < 0.01 and *p* < 0.05, respectively). In addition, some components of the ω3 group were also higher in adults, including C18:3 ω3 and C22:5 ω3 (*p* < 0.05), as well as long chain ω6 fatty acids, such as C20:3 ω6 and C22:2 ω6 (*p* < 0.01), and C20:4 ω6 (arachidonic acid). In contrast, subadults showed significantly higher levels of unsaturated fatty acids, particularly monounsaturated fatty acids such as C18:1 cis 9 (*p* < 0.05), and polyunsaturated fatty acids of the ω6 series, such as C18:2 cis ω6 (*p* < 0.05). Also noteworthy is the greater presence, always in subadults, of long‐chain saturated fatty acids such as C16:0 and C18:0 (*p* < 0.01 and *p* < 0.05, respectively).

**Table 2:** Comparison of the chemical composition and protein digestibility of *Acheta domesticus* according to stage of development.

	Subadults	Adults	RMSE	*p* value
DM, %	81.42^b^	93.17^a^	3.06	0.0101
Ash, % DM	7.82^A^	4.87^B^	0.24	0.0039
EE, % DM	28.09^A^	17.71^B^	1.09	0.0053
CP, % DM	50.02^B^	55.12^A^	2.94	0.0013
CF, % DM	10.48	12.16	1.12	0.3427
NFE, % DM	3.59^B^	10.14^A^	0.31	0.0002
GE, Kcal/Kg DM	6219.4^a^	5786.8^b^	329.65	0.0368
CPd, %	66.80^b^	68.57^a^	2.46	0.0292

*Note:* a, b = *p* < 0.05; A, B = *p *< 0.01.

Abbreviations: CP, crude protein; CPd, crude protein digestibility; DM, dry matter; EE, ether extract; GE: gross energy; NFE, Nitrogen‐Free Extract; RMSE, root mean square error.

**Table 3:** Level of toxic elements in *Acheta domesticus* samples (dry weight basis) according to the developing stage.

	Subadults	Adults	RMSE	*p*‐value
Cd, μg/kg	6.71^a^	5.03^b^	0.25	0.0201
Hg, μg/kg	6.11^A^	4.06^B^	0.19	0.0035
Pb, μg/kg	3.51	4.29	0.21	0.1562

*Note:* a, b: *p* < 0.05; A, B: *p* < 0.01.

Abbreviations: Cd, cadmium; Hg, mercury; Pb, lead.

**Table 4:** Levels Se, Fe, Ca, Mg, K and P measured in *Acheta domesticus* samples according to the developing stage.

	Subadults	Adults	RMSE	*p*‐value
Se, mg/kg	3.72^B^	5.51^A^	0.35	0.0017
Fe, mg/kg	0.384	0.365	0.032	0.0791
Ca, mg/kg	15.63	18.85	0.946	0.1054
Mg, mg/kg	8.90	8.73	0.3435	0.6052
K, mg/kg	113.3	111.4	8.83	0.6124
P, mg/kg	0.111^B^	0.144^A^	0.009	< 0.0001

*Note:* A, B: *p* < 0.01.

Abbreviations: Ca, calcium; Fe, iron; K, potassium; Mg, magnesium; P, phosphorus; RMSE, Root Mean Squared Error; Se, selenium.

**Table 5:** Fatty acid profile (% of FAME) of *Acheta domesticus* according to the stage of development.

	Subadults	Adults	RMSE	*p*–value
C4:0	6.71^b^	22.51^a^	5.083	0.0230
C6:0	1.57	0.00	1.51	0.2962
C8:0	0.13	0.00	0.23	0.5415
C10:0	3.87^b^	11.17^a^	1.92	0.0119
C12:0	1.45	2.58	1.13	0.3119
C14:0	0.88	1.44	0.61	0.3483
C14:1	0.12	0.00	0.083	0.1494
C16:0	26.98^A^	12.43^B^	2.10	0.0013
C16:1	0.41	0.00	0.25	0.1293
C17:0	0.13	0.00	0.22	0.5415
C17:1	0.13	0.00	0.08	0.1373
C18:1 cis 6	0.02	0.000	0.03	0.5415
C18:0	7.94^a^	4.49^b^	1.11	0.0234
C18:1 trans 9	1.30	1.41	0.36	0.7357
C18:1 cis 9	18.84^a^	10.07^b^	3.00	0.0280
C18:1 cis 11	0.36	0.57	0.29	0.4710
C18:2 trans ω6	0.00	1.52	0.02	< 0.0001
C18:2 cis ω6	24.41^a^	18.07^b^	2.38	0.0375
C20:0	0.31	0.42	0.35	0.727
C18:3 ω6	0.09^b^	0.93^a^	0.26	0.0229
C20:1	0.54	0.79	0.11	0.0578
C18:3 ω3	0.37^b^	1.20^a^	0.27	0.0199
C20:2 ω6	0.02	0.06	0.01	0.0628
C22:0	0.08^B^	0.19^A^	0.11	0.0076
C20:3 ω6	0.58^B^	4.16^A^	0.37	0.0004
C22:1	0.03	0.08	0.022	0.0652
C20:4 ω6	0.34^B^	1.71^A^	0.35	0.0101
C22:2 ω6	0.07^B^	0.40^A^	0.05	0.0030
C24:0	0.07^b^	0.20^a^	0.05	0.0489
C20:5 ω3	0.11	0.34	0.12	0.1033
C24:1	0.11	0.17	0.08	0.4532
C22:4 ω6	0.56	0.83	0.30	0.3524
C22:5 ω3	0.12^b^	0.38^a^	0.07	0.0187
C22:6 ω3	0.18	0.21	0.05	0.5245
SFA	50.15	55.48	3.16	0.1241
MUFA	21.91^a^	13.12^b^	2.73	0.0207
PUFA	26.84	29.88	2.00	0.1543
ω6	26.10	27.73	1.85	0.3667
ω3	0.73^b^	2.15^a^	0.49	0.0293
PUFA/SFA	0.53	0.53	0.06	0.9829
ω6/ω3	38.10^a^	13.10^b^	9.79	0.0464
LA/ALA	81.09^a^	16.63^b^	21.14	0.0244
AA/EPA	3.16	5.65	1.68	0.1621

*Note:* a, b = *p* < 0.05; A, B = *p *< 0.01.

Abbreviations: AA, Arachidonic acid; ALA, Alpha‐linolenic acid; EPA, Eicosapentaenoic acid; LA, linoleic acid; MUFA, monounsaturated fatty acids; PUFA, polyunsaturated fatty acids; RMSE, root mean square error; SFA, saturated fatty acids.

However, subadults also present a significant nutritional imbalance, with an extremely high ω6/ω3 ratio compared to adults (*p* < 0.05). The linoleic/α‐linolenic (LA/ALA) ratio, another key indicator of fat quality, was also strongly unbalanced in subadults (*p* < 0.05).

### 3.1 Chitin Extraction

The extraction process successfully removed the main non‐chitin components from all samples of subadults and adult cricket meal. The quantities of lipids and proteins removed, and the final chitin yield were determined gravimetrically and reported as a percentage of the initial dry weight of the sample in Table 6. The lipid content was significantly higher in subadults (25.95 ± 4.07%) compared to adult samples (14.21 ± 3.44%) (unpaired *t*‐test, *t* = 6.231, *df* = 14, *p* < 0.0001). Protein content was similar between the two stages (SA: 39.76 ± 8.74%; A: 45.06 ± 5.83%). Although adults tended to have a higher mean protein percentage, the difference was not statistically significant. Chitin content showed a slight, non‐significant increase in adults compared with subadults. The analysis of correlation between chitin content and protein digestibility of cricket samples revealed a high, negative relationship between the two parameters. The regression equation describing the relationship between chitin content (%) and crude protein digestibility (CPd, %) was: CPd (%) = 72.74 − 3.42 × chitin (%), R^2^ = 0.61, *p* < 0.001.

**Table 6:** Percentages of lipids and proteins removed from subadult and adult *Acheta domesticus* flour, and chitin extraction yield.

	Subadults	Adults	RMSE	*p*‐value
Lipids, %	25.95 ± 4.07^A^	14.21 ± 3.44^B^	4.20	0.0001
Proteins, %	39.76 ± 8.74	45.06 ± 5.83	8.09	0.3104
Chitin, %	3.62	4.97	1.76	0.1632

*Note:* A, B: *p* < 0.01.

Abbreviation: RMSE: root mean square error.

### 3.2 ATR‐FTIR Analysis

**Figure 1:**
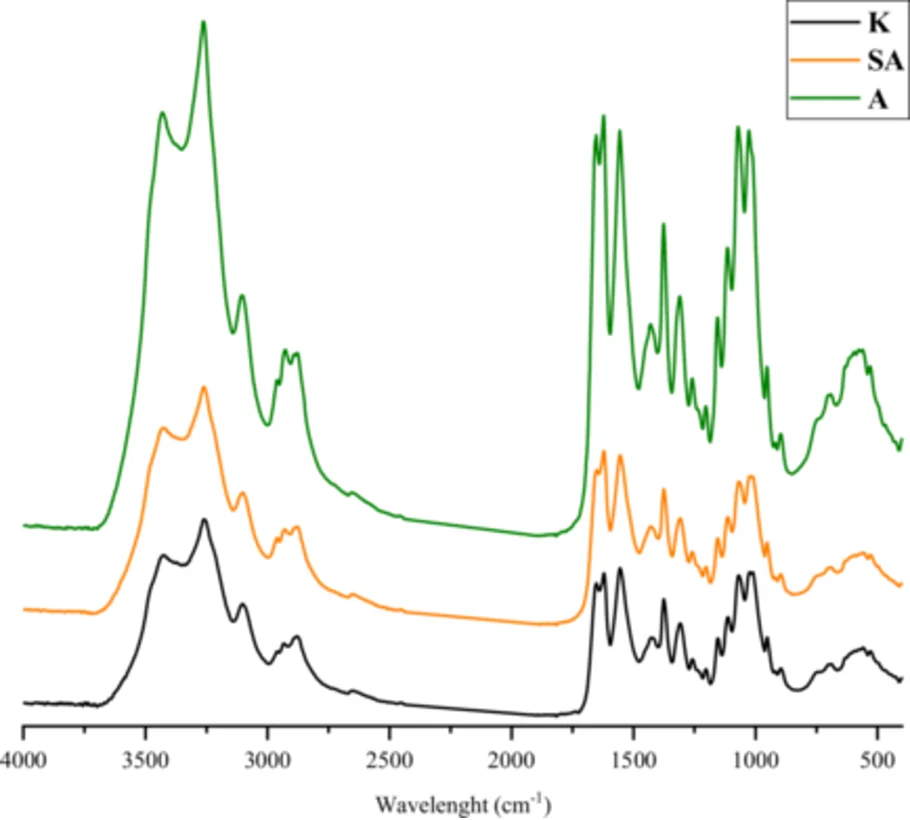
ATR‐FTIR spectra of chitin extracted from *Acheta domesticus*. For spectra comparison, subadult (SA – orange line), adult (A – green line), and commercial chitin (K – black line) samples are reported. [Color figure can be viewed at wileyonlinelibrary.com]

The ATR‐FTIR spectra of chitin samples obtained from *A. domesticus* are presented in Figure 1. In all spectra, the characteristic chitin absorption bands are clearly visible. As for commercial chitin spectrum (K), both adults and subadults samples exhibit amide III and amide II bands (around 1310–1320 cm^−1^ and 1550–1560 cm^−1^, respectively), CO‐stretching signal at 1650–1655 cm^−1^ and OH‐stretching at 3430–3450 cm^−1^, confirming the identification of an α‐chitin structure (Triunfo et al. 2022; Robinson et al. 2022; Khayrova et al. 2020; Purkayastha and Sarkar 2020; Nandiyanto et al. 2019).

## 4 Discussion

The analysis of the chemical composition and protein digestibility of *Acheta domesticus* highlighted a clear influence of the insect life cycle on the nutritional quality of the final product. The dry matter content (% DM) was significantly higher in adults than in subadults, confirming that the age of the insect substantially influences the nutrient concentration (Pedrazzani et al. 2024). Regarding the ether extract (% EE), significantly higher values in subadults than in adults could indicate a specific physiological and metabolic role of lipids in this developmental phase, related to the preparation for moulting or to the accumulation of energy necessary for growth, while their reduction in adults could result from a greater metabolic use or a different allocation of nutrients in tissues (Magara et al. 2021). From a nutritional point of view, a higher level of lipids is responsible for a high gross energy level in SA. This trend has been observed in several insect species, although exact values and trends may vary depending on species, rearing conditions, and diet (Manzano‐Agugliaro et al. 2012). The percentage of lipids measured in adults and subadults allows us to make two considerations: firstly, the SA appear to be better candidates than adults for oil extraction; secondly, they are more difficult than adults to include in whole form in animal diets, for technological and nutritional concerns. The crude protein (% CP) content, significantly higher in adults than in subadults, suggests increased protein synthesis in mature individuals, likely due to the complete formation of the muscular and cuticular apparatus, which represents a substantial portion of the insect's body mass (Mariod et al. 2017). Crude protein digestibility (% CPd) measured in vitro, with significantly higher values in adults, suggests not only a higher protein content but also a potentially improved protein quality. In general, differences in protein digestibility have been associated with variations in amino acid composition and the presence of antinutritional factors, which are known to affect protein hydrolysis and amino acid availability in food proteins and insect meals (Rumpold and Schlüter 2013; Sarwar Gilani et al. 2012). In the literature, in vitro CPd of *A. domesticus* shows considerable variability, depending on the analytical methodology, sample treatment, and structural constraints related to chitin. While some studies report very high values (Hammer et al. 2023), other authors describe a much wider range of results. For example, Rodríguez‐Rodríguez et al. (2024) highlighted that values in the literature can vary widely, from 57% to 91.7% (Bosch et al. 2014; Kovitvadhi et al. 2019). In this context, the digestibility values obtained in our study (66.80% in subadults and 68.57% in adults) fall within the lower but still documented range for *A. domesticus*. Although adults showed slightly higher mean chitin values than subadults, the high within‐group variability observed in our samples prevented this difference from reaching statistical significance. This variability is consistent with previous evidence indicating that chitin content and yield can be strongly influenced by individual factors, including developmental stage and physiological status. Indeed, Pedrazzani et al. (2024) demonstrated that chitin yield in crickets is influenced by killing method and specific developmental stage, while differences in meal composition and structural organisation during the transition from subadults to adult further contribute to variability in chitin extraction efficiency (Finke 2007; Van Peer et al. 2024). The presence in *A. domesticus* adult of sexually dimorphic sclerotized structures, such as pseudo elytra in males and the ovipositor in females (Kulma et al. 2019), has been shown to increase the structural fraction of the exoskeleton. However, the marked individual variability observed within groups likely masked any potential effect of these traits, preventing them from emerging as statistically detectable differences. Consistent with this, Espinosa‐Solís et al. (2024) reported higher chitin yields in adult crickets compared to nymphs, likely due to reduced protein binding to chitin. The chitin yields obtained in the present study generally fell within the ranges reported in the literature (4%–20%, depending on extraction conditions), although lower values were observed in some specimens, primarily subadults, consistent with previous findings (Carolyne et al. 2017; Espinosa‐Solís et al. 2024). Despite the absence of significant differences in chitin concentration among the experimental groups, the statistical analysis revealed a pronounced and highly significant inverse relationship between chitin content and CPd. This association supports the hypothesis that chitin may limit protein utilisation from insect‐based substrates. In agreement with this interpretation, recent studies have reported a consistent negative correlation between acid detergent fibre (ADF) and protein digestibility indices in insect meals (Rodríguez‐Rodríguez et al. 2024). In insect‐derived materials, ADF is commonly considered a proxy of the chitin‐rich structural fraction, which may also include proteins tightly bound to chitin within the cuticular complex (Finke 2007; Marono et al. 2015). Therefore, the accumulation of chitin and chitin–protein complexes may contribute to the reduction in protein digestibility, particularly when insect meals are included at relatively high levels in animal diets (Bovera et al. 2018). Regarding the mineral profile, the results of the present study indicate that *A. domesticus* meals from subadults and adults generally contain low levels of heavy metals. Although statistically significant differences in Cd and Hg concentrations were observed between subadults and adult samples, all measured values remained well below the maximum levels established by European legislation for feed materials: 0.5 mg/kg for cadmium, 5.0 mg/kg for lead, 0.1 mg/kg for mercury and 2.0 mg/kg for arsenic (Commission Regulation (EU) 1869/2019; Commission Regulation (EU) 744/2012; Commission Regulation (EU) 1275/2013; Directive (EC) 2002/32). Therefore, these differences should be interpreted as reflecting stage‐related variability in accumulation dynamics rather than indicating any toxicological concern. Heavy metal accumulation in edible insects is primarily influenced by the rearing substrate, which represents the main source of contamination. Therefore, the differences observed between developmental stages in this study are more likely related to physiological and growth‐related factors rather than species‐specific accumulation mechanisms. The observed concentrations of Hg and Cd highlight the need for regular monitoring and control measures to ensure the safety of this flour for animal consumption, given the toxicological risks associated with these metals. Similar stage‐dependent patterns in the accumulation of potentially toxic elements have been reported in other insect species and have been attributed to differences in accumulation dynamics and growth‐related dilution effects during development (Charlton et al. 2015; Poma et al. 2017). At the request of the European Commission, the EFSA Panel on Nutrition, Novel Foods and Food Allergens, was asked to provide an opinion on dried cricket powder (*A. domesticus*) as a novel food under Commission Regulation (EU) 2015/2283 (Commission Regulation (2015/2283). The Panel found that the level of contaminants in cricket meals depends on the presence of these substances in the insect feed (EFSA 2022). However, further studies with a larger sample size and the inclusion of analysis for other pollutants are essential. Concerning the concentrations of Pb, Cd, Hg and As, these preliminary results indicate that the tested samples can be used as animal feed without additional risks. The concentrations of heavy metals detected in the present study were generally lower than or similar to those reported in the literature for *A. domesticus* (Ajdini et al. 2025; Ramos et al. 2024).

In the present study, cadmium levels ranged from 0.005 to 0.007 mg/kg, which are markedly lower than those reported by Ajdini et al. (2025) (0.069–0.127 mg/kg) and Ramos et al. (2024) (0.134 mg/kg). Similarly, lead concentrations (0.0035–0.0043 mg/kg) were substantially lower than the values reported in both studies (0.05–0.12 mg/kg and 0.1 mg/kg, respectively).

Mercury levels (0.0041–0.0061 mg/kg) were comparable to those reported by Ajdini et al. (2025) (0.0065–0.0141 mg/kg) and slightly higher than the values reported by Ramos et al. (2024) (< 0.005 mg/kg). Arsenic was below the limit of quantification in all samples in the present study, whereas it was detected in both Ajdini et al. (2025) (0.08–0.36 mg/kg) and Ramos et al. (2024) (0.025 mg/kg). In addition to safety considerations related to heavy metals, other components of the mineral profile also showed significant differences between the different developmental stages. In particular, selenium and phosphorus were significantly higher in adults compared to subadults. Although specific selenium concentrations in crickets are highly dependent on both species and husbandry conditions, several studies demonstrate that the overall mineral content of edible insects is highly variable and influenced by diet and physiological development (Finke 2015). Furthermore, edible crickets have been reported as a source of essential minerals (Murugu et al. 2021). However, the actual nutritional contribution of these minerals in animal feeding depends on their inclusion rate in the diet and the specific nutritional requirements of the target species. Regarding phosphorus, the higher levels observed in adult crickets may reflect the increased accumulation of biomolecules associated with full somatic maturation. Insects store phosphorus primarily in the form of phosphate‐containing compounds such as phospholipids, nucleic acids, ATP, and phosphoproteins (Chapman 2013), all of which are critical components of membrane structure, cell signalling, and energy metabolism. This physiological pattern is consistent with the increased metabolic activity and structural consolidation that occur in later developmental stages. Studies on insect‐based ingredients, such as black soldier fly larvae meals, show efficient mineral utilisation and high overall nutrient digestibility (Schiavone et al. 2017). These findings support the potential role of *A. domesticus* meals as valuable mineral sources. The analysis of the fatty acid profile of *A. domesticus* revealed significant differences due to the developmental stage. In general, adults present a higher level of saturated fatty acids. Our data are consistent with the data of Ajdini et al. (2025) and Orkusz et al. (2023), who state that adults of *A. domesticus* present a relatively high percentage of saturated fatty acids. On the contrary, subadults show a higher presence of monounsaturated fatty acids, compared to adults. This suggests a greater plasticity of cell membranes and a more active metabolism (Perez‐Santaescolastica et al. 2023). It was found that some fatty acids are strongly influenced by the rearing substrate, highlighting that environmental or dietary factors can substantially modify the lipid profile, with sometimes very marked differences even at the same developmental stage. Adding omega‐3‐rich ingredients to the diet, such as algae or flaxseed oil, can substantially improve the lipid profile, reducing imbalances between different fatty acid classes and making crickets a healthier source of protein and lipid for animal feed (Oonincx et al. 2019; Ajdini et al. 2024 2025). Furthermore, the analysis of the ratios between different lipid classes, such as ω6/ω3 and LA/ALA, revealed significant imbalances that suggest nutritional implications.

However, a higher proportion of unsaturated fatty acids should not be considered universally advantageous in livestock nutrition, as it may reduce the oxidative stability of animal‐derived products and negatively affect shelf life, technological properties, and sensory quality. Therefore, the relevance of this lipid profile depends on the intended application being potentially more suitable for pet food or specific nutritional purposes than for livestock production where product stability is a major concern.

Indeed, ω6/ω3 ratios can be unbalanced depending on the rearing conditions and developmental stage (Orkusz et al. 2023; Perez‐Santaescolastica et al. 2023; Paul et al. 2017). These results highlight the importance of standardising rearing conditions to ensure a more balanced and consistent nutritional composition, especially if the insect is used in areas where lipid quality is a critical parameter. The significantly higher ω6/ω3 ratio observed in subadults can be interpreted in light of physiological differences between developmental stages. According to FAO (2013), the nutritional profile of insects is strongly influenced by the metamorphic stage, with growing individuals generally presenting higher proportions of unsaturated fatty acids, particularly PUFAs such as linoleic acid (ω6), which are abundant in rapidly developing tissues. In contrast, adults presented higher proportions of saturated fatty acids and a more balanced ω6/ω3 ratio. Saturated fatty acids, which confer greater membrane rigidity and oxidative stability, tend to accumulate in later developmental stages, when metabolic turnover is lower and structural consolidation is completed (FAO 2013). This stage‐dependent pattern is further contextualised by the fatty acid profile of the feed used in the present study. In this context, the lipid profile of subadult crickets appears to more closely reflect the fatty acid composition of the diet, particularly in terms of the relative abundance of linoleic acid and total ω6 fatty acids, whereas adult individuals show a partial divergence from the feed profile, likely due to stage‐dependent metabolic remodelling. Furthermore, the presence of long‐chain ω6 fatty acids such as arachidonic acid (C20:4 ω6) in *A. domesticus* may be partly explained by the insect's ability to biosynthesize these compounds *de novo*. These findings suggest that developmental stage may influence not only the amount, but also the biological quality, of lipids in *A. domesticus* intended for feed use (Jurenka et al. 1988; Hasan et al. 2019; Zhou et al. 2008).

## 5 Conclusion

The results of the present study confirm that the developmental stage markedly affects the nutritional characteristics of *A. domesticus* meal. Adult crickets showed higher CP content and greater in vitro CPd than subadults, indicating a better protein‐oriented nutritional profile. These findings suggest that adult crickets may be more suitable when protein quality and digestibility are the main criteria for feed formulation.

Subadult crickets, instead, were characterised by higher ether extract content and gross energy value. However, this should not be interpreted as an overall nutritional advantage, since lipid quality and the intended use of the ingredient must be carefully considered. In particular, the higher proportion of unsaturated fatty acids may be relevant for specific applications, such as pet food or energy‐oriented formulations, but may also raise concerns in livestock production due to possible effects on oxidative stability, shelf life, and sensory quality of animal‐derived products.

Chitin content did not differ significantly between developmental stages, although its negative relationship with CPd suggests that the structural fraction of insect meals may influence protein availability. Mineral analysis showed stage‐related differences, particularly for selenium and phosphorus, while the concentrations of heavy metals remained below the regulatory limits for feed materials, supporting the safety of the tested samples.

Overall, adult and subadult *A. domesticus* meals should be considered nutritionally distinct ingredients rather than interchangeable products. The choice of the developmental stage should therefore depend on the intended feed application, with adults being more appropriate for protein‐oriented uses and subadults potentially more suitable for lipid‐ or energy‐oriented purposes.

## Author Contributions

Evolveat Company provided the *Acheta domesticus* specimens used in this study but had no role in the study design, data collection and analysis, interpretation of results, decision to publish, or preparation of the manuscript.

## Funding

The authors have nothing to report.

## Ethics Statement

The authors confirm that the study complied with the ethical policies of the journal. No vertebrate animals were used. *Acheta domesticus* (invertebrates) were obtained from a commercial producer and were handled and processed according to good practice to minimise stress; harvesting/euthanasia was performed by rapid chilling/freezing, as described in the Methods. Therefore, no institutional animal ethics approval was required under applicable regulations.

## Conflicts of Interest

The authors declare no conflicts of interest.

## Data Availability

The data that support the findings of this study are available on request from the corresponding author. The data are not publicly available due to privacy or ethical restrictions.
